# Physical correlates of human-like softness elicit high tactile pleasantness

**DOI:** 10.1038/s41598-021-96044-w

**Published:** 2021-08-13

**Authors:** Ryo Kitada, Megan Ng, Zheng Yee Tan, Xue Er Lee, Takanori Kochiyama

**Affiliations:** 1 Division of Psychology, School of Social Sciences, Nanyang Technological University, 48 Nanyang Avenue, Singapore, 639818 Singapore; 2 Graduate School of Intercultural Studies, Kobe University, 1 Chome-2-1 Tsurukabuto, Nada Ward, Kobe, Hyogo 657-0013 Japan; 3 ATR-Promotions, Brain Activity Imaging Center, 2-2-2 Hikaridai Seika-cho, Sorakugun, Kyoto, 619-0288 Japan

**Keywords:** Human behaviour, Somatosensory system

## Abstract

Touching an object can elicit affective sensations. Because these sensations are critical for social interaction, tactile preferences may be adapted to the characteristics of the human body. We have previously shown that compliance, a physical correlate of softness, increased the tactile pleasantness of a deformable surface. However, the extent to which object compliance similar to the human body elicits tactile pleasantness remains unknown. We addressed this question by using a wide range of compliances and by measuring the distribution of compliance of human body parts. The participants numerically estimated the perceived pleasantness or softness while pushing tactile stimuli with their right index fingers. The perceived softness monotonically increased with increasing compliance and then leveled off around the end of the stimulus range. By contrast, pleasantness showed an inverse U pattern as a function of compliance, reaching the maximum between 5 and 7 mm/N. This range of compliance was within that for both hand and arm. These results indicate that objects with similar compliance levels as those of human body parts yield the highest pleasantness when pushing them.

## Introduction

When touching an object, we not only perceive its physical properties but also experience affective sensations associated with them. While the former is called discriminative touch, the latter is affective touch. Previous studies suggest that affective touch is critical not only for cognitive and emotional development in childhood but for a general sense of well-being in adulthood^1–6^. For instance, being touched by close friends or partners can alleviate the distress of physical and social pain^7,8^. The bodily area that human adults allow others to touch is associated with an emotional bond, a key index of social networks across cultures^9,10^. These findings raise the possibility that, to satisfy our essential need for social bonds, our preferences for touch are adapted to the physical properties of the human skin^11^. Though the psychophysical attributes of affective touch have been investigated, its nature and underlying mechanisms are not fully understood.

One way to investigate the psychophysical attributes of affective touch is to examine the extent to which the physical attributes associated with discriminative touch can influence affective touch. Tangible object properties are categorized into macro-geometrical (e.g., shape) and material properties (e.g., softness). Among material properties, roughness, softness, and temperature are considered prominent perceptual dimensions of discriminative touch and account for the perceptual space of an object's surface^12,13^. Valence (pleasantness and comfort) and arousal can account for the perceptual dimensions of affective touch^14,15^, consistent with Russell’s circumplex model^16^. A separate area of research compared affective touch dimensions with dimensions that contribute to discriminative touch by using multivariate analyses on ratings of adjectives^14,15,17^. Another line of research compared patterns of valence with patterns of perceived roughness when the subjects touched the same objects^18–20^. These studies demonstrated that valence was related to perceived roughness. However, these studies did not examine how the valence pattern is related to the material properties of the human body.

If tactile preferences are adapted to the human skin and body, then we can expect that the physical properties of the skin and body parts should be close to the physical properties that yield the highest pleasantness. In accordance with this hypothesis, previous studies demonstrated that under normal thermal conditions, the most pleasant temperature was that close to our body temperature^21,22^. However, it is unknown if the same hypothesis can be applied to other perceptual dimensions of material properties such as softness.

Previous studies have examined physical factors that determine the tactile perception of softness and demonstrated that perceived softness increases as a function of object compliance, defined as the magnitude of deformation of an object under applied force^23,24^. Pasqualotto et al. (2020) examined the association between perceived softness and pleasantness by varying compliance of objects^11^. More specifically, our previous study showed that the magnitude of pleasantness increased as a function of compliance^11^. However, because the range of compliance was limited, it is still unclear whether pleasantness keeps increasing at higher compliance or shows an inverted U-shape pattern with the peak pleasantness at the corresponding compliances for the human body.

The objective of the present study was to examine the range of compliance that yields the highest pleasantness of deformable surfaces. We used the same set of stimuli from our previous study^11^ with an increased range of compliance. We collected the human hand and forearm compliance to test the hypothesis that pleasantness reaches a plateau within the range of compliance for the hand and forearms and decreases with higher compliance.

## Results

**Figure 1:**
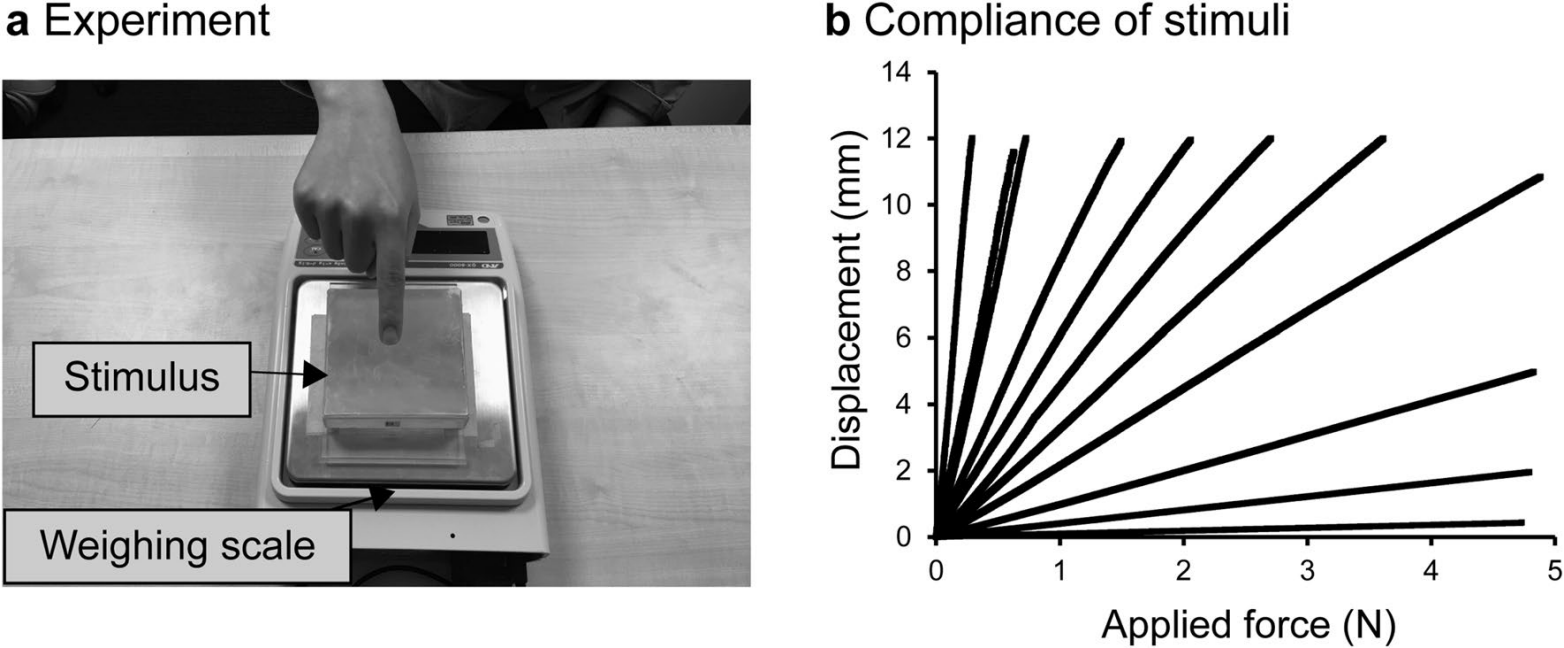
Stimuli and stimulus presentation (Experiment 1). (**a**) *Task* Two groups of healthy volunteers (24 for each) participated in the experiment. The participants in both groups used their right index fingers to touch the same location (e.g., center) of each stimulus on the scale. After touching the surface up to three times, half of the participants were asked to estimate pleasantness (pleasantness-instruction group), whereas the other half were asked to estimate softness (softness-instruction group). Data were normalized and log transformed (base 10). The scale transferred the applied vertical force data to the computer (not shown). (**b**) *Stimulus and compliance* All stimuli were made of urethane rubbers of varying compliance. The relationship between applied force and displacement was measured for each stimulus with a compression tester. Compliance of each stimulus was defined as the slope of the linear function fitted to displacement as a function of applied force.

We conducted two types of experiments in the present study. The purpose of the first was to obtain magnitude estimates of perceived softness and pleasantness. The two groups of participants (24/group) touched deformable surfaces made of urethane rubber with their right index fingers (Fig. 1a). Compliance (a physical correlate of softness) differed among stimuli (Fig. 1b). Compliance values for plastic, wood, and foamed styrol are < 1 mm/N, whereas the compliance of a sponge is roughly over 2 mm/N^25^. One group of participants was instructed to estimate the perceived softness, whereas the other was asked to report pleasantness (absolute magnitude estimation procedure^26^). The obtained values (magnitude estimates) were normalized and transformed into logarithms (base 10)^26,27^. Contact duration and the maximum force exerted by the finger were recorded using a weighing scale. In the second experiment, we measured the distribution of compliance of the forearm and hand from 70 participants to examine the relationship between the most pleasant compliance and that of body parts.

### Patterns of mean magnitude estimates

Initially, we examined the mean of magnitude estimates as a function of compliance to test whether there were any peak estimates in pleasantness. The softness-evaluation (henceforth softness) group served as a control.

### Pleasantness

**Figure 2:**
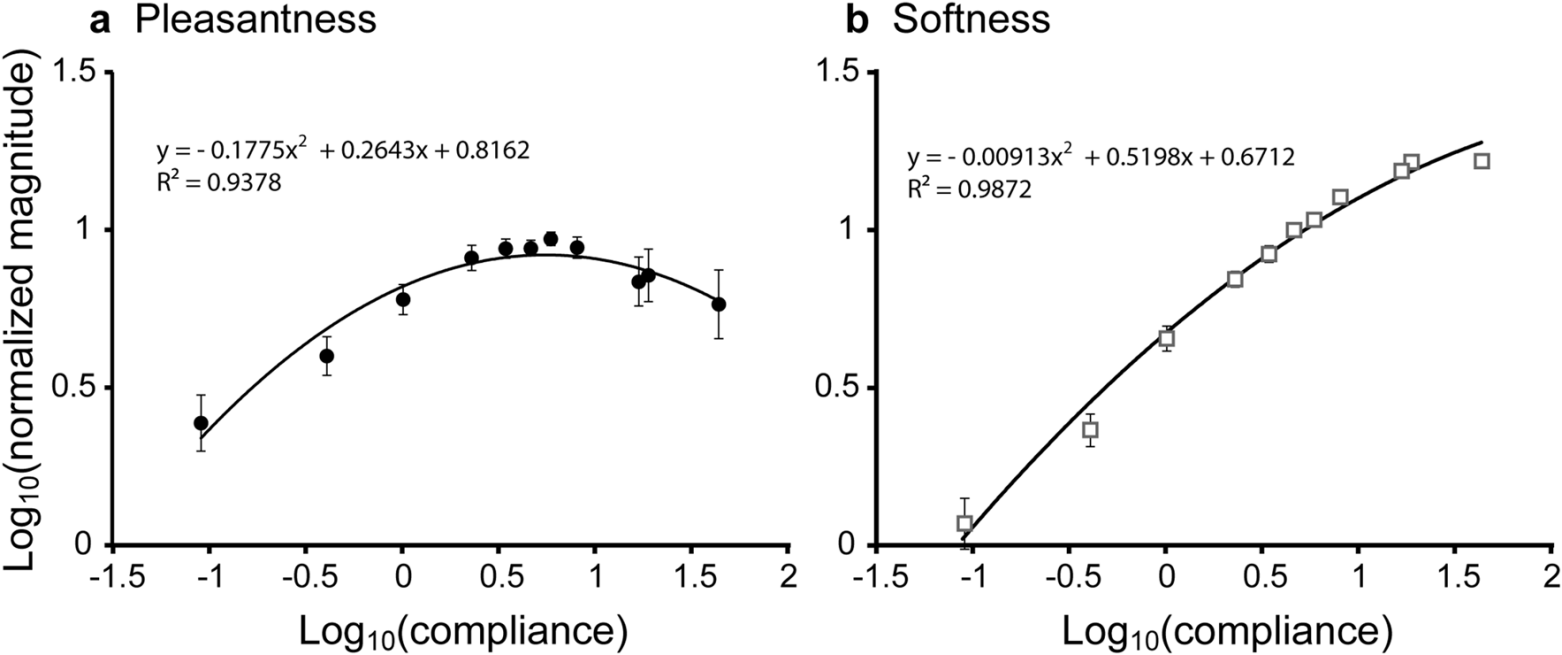
Magnitude estimates of pleasantness and softness. Mean log_10_ normalized magnitude estimates of pleasantness and perceived softness plotted as a function of log_10_(compliance). Each data point indicates the mean ± standard error of the mean (SEM) of 24 participants. Note that the two groups of participants in each experiment evaluated softness and pleasantness separately.

Figure 2a shows pleasantness as a function of compliance on base-10 logarithmic scales. The mean pleasantness monotonically increased and gradually reached a plateau at compliance values from 3.39 mm/N (0.53 on a base-10 logarithmic scale) to 8.06 mm/N (0.91) and then decreased.

To statistically test if pleasantness follows an inverted U-shape pattern, we performed linear mixed model (LMM) analyses on pleasantness. We did not use a conventional repeated-measures ANOVA because of the different variances among stimuli. A higher-order polynomial function can account for the pattern of observed pleasantness estimates, while such a function may be overfit to the data. We assumed the following two models. The first only included a linear trend assuming a linear function, while the second included quadratic and linear trends to account for any inverted U-shape pattern. The model selection criteria (Bayesian information criterion [BIC]) preferred the quadratic model to the linear one (∆BIC = 20.1), indicating that the first is more appropriate than the second. The LMM with the quadratic model showed that both quadratic and linear trends were significant [*F*(1, 45.2) = 72.5, *P* < 0.001 for the linear trend; *F*(1, 85.3) = 29.1, *P* < 0.001 for the quadratic trend]. Since the quadratic trend was significant, we fitted the quadratic function to the pleasantness data. The R^2^ value for the fitted quadratic function was around 0.94. The peak of the fitted function was located at 5.6 mm/N (0.74 on the log scale). These results confirm that the quadratic model well accounted for variances of pleasantness. Moreover, the compliance value that provided the highest pleasantness was within the compliance values of the stimuli.

### Softness

Figure 2b shows perceived softness as a function of compliance on base-10 logarithmic scales. The perceived softness monotonically increased with increasing compliance, leveling off at the upper end. We performed the same LMM analyses on the softness. The Bayesian information criterion indicated that the model with quadratic and linear trends was better than that, including only a linear trend (∆BIC = 59.6). The LMM with the quadratic model showed that the quadratic trend and linear trend were both significant [*F*(1, 88.1) = 426.8, *P* < 0.001 for the linear trend; *F*(1, 105.8) = 111.81, *P* < 0.001 for the quadratic trend]. Since the quadratic trend was significant, we fitted the quadratic function to softness magnitude estimates. R^2^ values for the fitted quadratic function were around 0.99. The peak of the fitted function was located at 702.5 mm/N (2.84 on the log scale), well beyond the range of compliance values of stimuli. These results indicate that the magnitude estimate of perceived softness monotonically increased as a function of compliance within the stimulus range, though the quadratic trend accounts for the variance.

### Variances of magnitude estimates

Figure 2 shows that standard errors of the mean (shown as error bars) also differed among stimuli. Thus, we conducted an exploratory analysis to examine the pattern of variances of magnitude estimates as a function of compliance.

**Figure 3:**
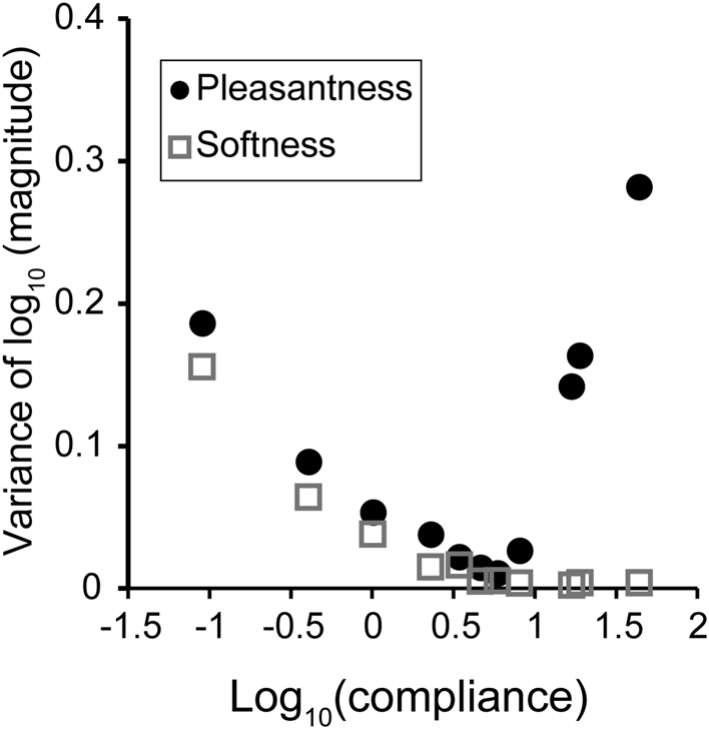
Variance of magnitude estimates. Variances of log_10_ magnitude estimates were plotted as a function of log_10_ compliance.

Figure 3 shows the variances of the softness and pleasantness estimates. Both patterns of variances differed. More specifically, variances for pleasantness showed a U-shaped pattern, and the minimum variance was observed at the compliance value of 0.77. This compliance value (5.9 mm/N) was close to that at the pleasantness peak (5.6 mm/N, Fig. 2a). In contrast, the variances for perceived softness decreased as a function of compliance.

To statistically evaluate different patterns of variances among stimuli, we performed Levene's tests for equality of variances on softness variances (with the correction of false-discovery rate). The variances for the stimuli which provided high mean pleasantness (0.53, 0.66, 0.77, and 0.91 on the logarithmic scale) were significantly smaller than variances for the least compliant (hardest) stimuli (− 1.06 and − 0.39) and the most compliant (softest) stimuli (1.23, 1.28, and 1.64) (*p* values < 0.05). The same tests on softness showed that variances for the least compliant stimuli (-1.06, -0.39, and 0.02) were significantly greater than those for more compliant stimuli (each stimulus from 0.67 to 1.64) (*p* values < 0.05, details of statistical results are available in Supplementary Table 1).

Collectively, pleasantness variances showed a U-shaped pattern as a function of compliance with the minimum variance located around the compliance value that yielded the highest mean pleasantness. In contrast, such a pattern was not observed in softness; softness variances tended to decrease as a function of compliance. This result indicates that individual estimate differences are smallest at compliance values with the highest mean pleasantness.

### Individual differences in most pleasant compliance

Although the peak pleasantness was located at 5.6 mm/N (0.74 on the log scale) in the group data (Fig. 2a), individual differences were noted in the compliance yielding the highest pleasantness. To examine such individual differences, we calculated the compliance that provides the highest pleasantness for each participant by fitting the quadratic function to *individual* data. The assumption of this analysis is that the fitted quadratic function of each participant is convex; a compliance value providing the highest magnitude estimate should correspond with the peak. Therefore, we excluded the data of individuals in which the fitted quadratic function was concave (three participants’ data from softness data and one from pleasantness data).

**Figure 4:**
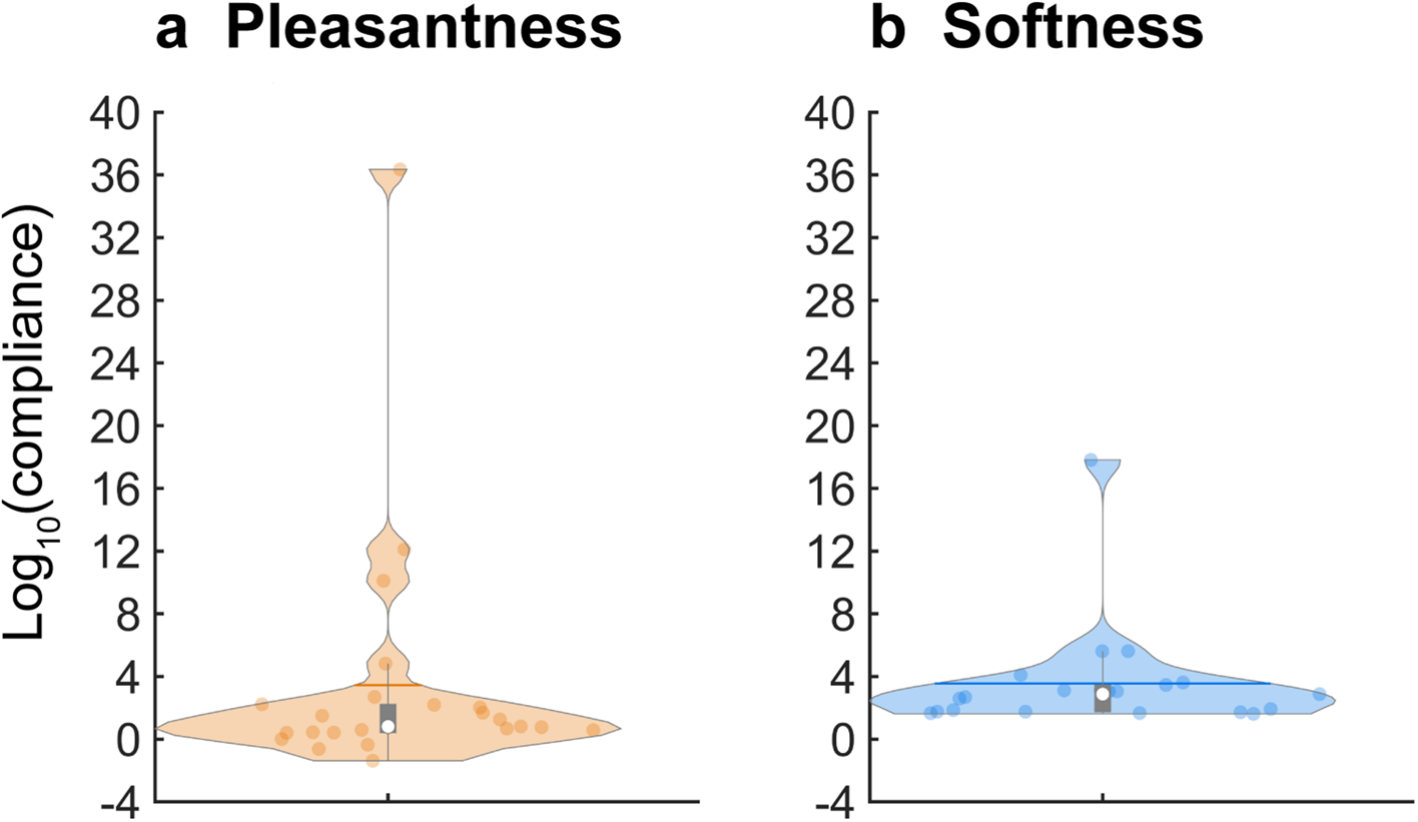
Distribution of compliance values providing the highest magnitude estimate. Distributions of log_10_ compliance values providing the highest estimates for pleasantness (**a**) and softness (**b**) are shown as violin plots. Each dot represents the individual participant’s compliance providing the highest pleasantness (**a**) and the highest softness (**b**). The white circle, thick horizontal line, and bar indicate the median, mean, and box plot, respectively. The medians for pleasantness and softness were 0.81 (6.5 mm/N) and 2.87 (752.8 mm/N), respectively.

Figure 4 shows the distribution of the log_10_ compliance values that produced the highest magnitude estimate in pleasantness and softness, respectively. Compliance values at the estimates’ peak ranged from -1.4 to 36.3 (on a base-10 logarithmic scale) for pleasantness and 1.6 to 17.8 for softness. We conducted non-parametric analysis because Kolmogorov–Smirnov tests showed that the data were not normally distributed (p values < 0.01, Bonferroni-corrected). The medians for softness and pleasantness were 2.87 (752.8 mm/N) and 0.81 (6.5 mm/N), respectively. The Mann–Whitney U test showed significantly different distributions for the pleasantness and softness groups (*P* = 0.003). Taken together, though peak compliance values varied among participants, the median compliance value for pleasantness was close to the peak group-mean compliance (5.6 mm/N, 0.74 on the log scale).

Finally, because autistic traits can be associated with atypical tactile sensitivity^28^, we also examined to what extent the Glasgow Sensory Questionnaire^29^ (GSQ) can explain individual differences in fitted functions (peak compliance value for pleasantness and softness). However, no significant correlation was observed between the GSQ and peak compliance values (Spearman' rho = 0.18 for pleasantness; rho = − 0.20 for softness).

### Did the participants report softness instead of pleasantness?

We observed a monotonic increase in pleasantness as a function of compliance in some participants, shown as high compliance in Fig. 4a. Because perceived softness tends to monotonically increase with compliance, this raises the possibility that some participants may have simply reported on physical characteristics (softness) instead of pleasantness^11^. If this were the case, they are likely to apply the same heuristics to other stimuli. To examine this point, we conducted an experiment where participants in the pleasantness group estimated pleasantness when touching surfaces consisting of various numbers of spherical segments (Supplemental Fig. 1). Since the spherical segments were made of the same material, pleasantness should not monotonically increase with the number of segments on the surface. However, the pleasantness of five participants increased together with the number of spherical segments, indicating that these participants may have reported softness instead of pleasantness.

Thus, we reanalyzed the data after excluding these five participants. Nevertheless, we confirmed the inverted U-shaped pattern of log_10_ pleasantness (Supplementary Fig. 2). More importantly, the fitted quadratic function indicated that the maximum pleasantness was observed at log_10_ compliance value 0.69 (corresponding to 4.9 mm/N), close to the compliance found when all data were included in the analysis (5.6 mm/N, Fig. 2a). Thus, it is unlikely that the overall pattern of pleasantness was affected by the participants who reported softness instead of pleasantness.

### Maximum force applied with the finger and contact duration

**Figure 5:**
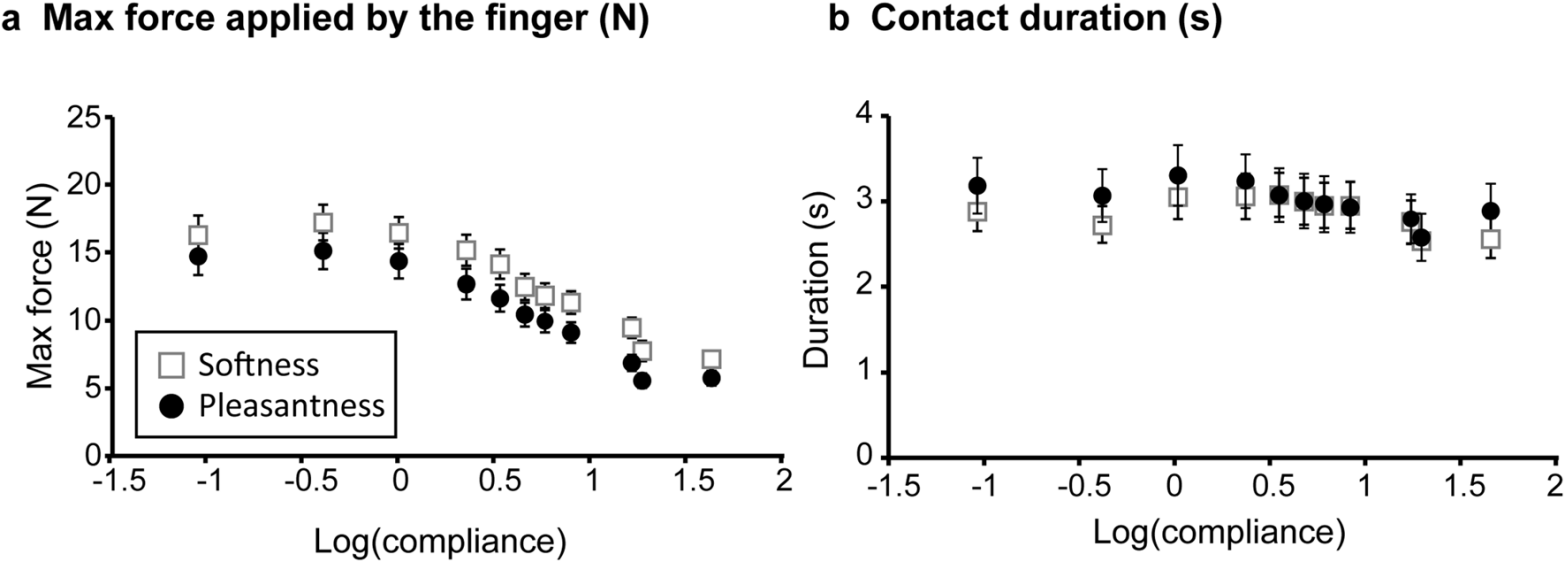
Maximum force and contact duration. Patterns of maximum force applied by the finger and contact duration plotted as a function of log_10_(compliance). Each data point indicates the mean ± standard error of the mean (SEM) of 24 participants.

Figure 5a shows the mean maximum force exerted by the finger when participants pushed the stimuli. Maximum force decreased as a function of compliance. We performed LMM analysis on the maximum force with a model that includes linear and quadratic trends of compliance and group (pleasantness and softness) as fixed-effect factors. This analysis showed significant effects for the linear trend [*F*(1, 42.9) = 20.7, *P* < 0.001] and for the quadratic trend [*F*(1, 217.1) = 107.7, *P* < 0.001]. The effect of group was also significant [*F*(1, 46.8) = 4.08, *P* = 0.049]. No other effect was significant (*p* values > 0.1). Like magnitude estimates, we fitted the quadratic function to maximum force. R^2^ values for the fitted quadratic function were around 0.95 for pleasantness and 0.96 for softness. The fitted quadratic functions were convex in both instruction groups; coefficients for the quadratic terms were -1.52 for pleasantness and -1.90 for softness. Compliance values at the highest force were -1.05 for pleasantness and -0.77 for softness (on a logarithmic scale). This result confirms that the maximum force in both instruction groups tended to decrease as a function of compliance. In contrast, contact duration was highly similar across stimuli, despite some effects of compliance (Fig. 5b) (see Supplementary results for details on statistical analyses).

### Human forearm and hand compliance

**Figure 6:**
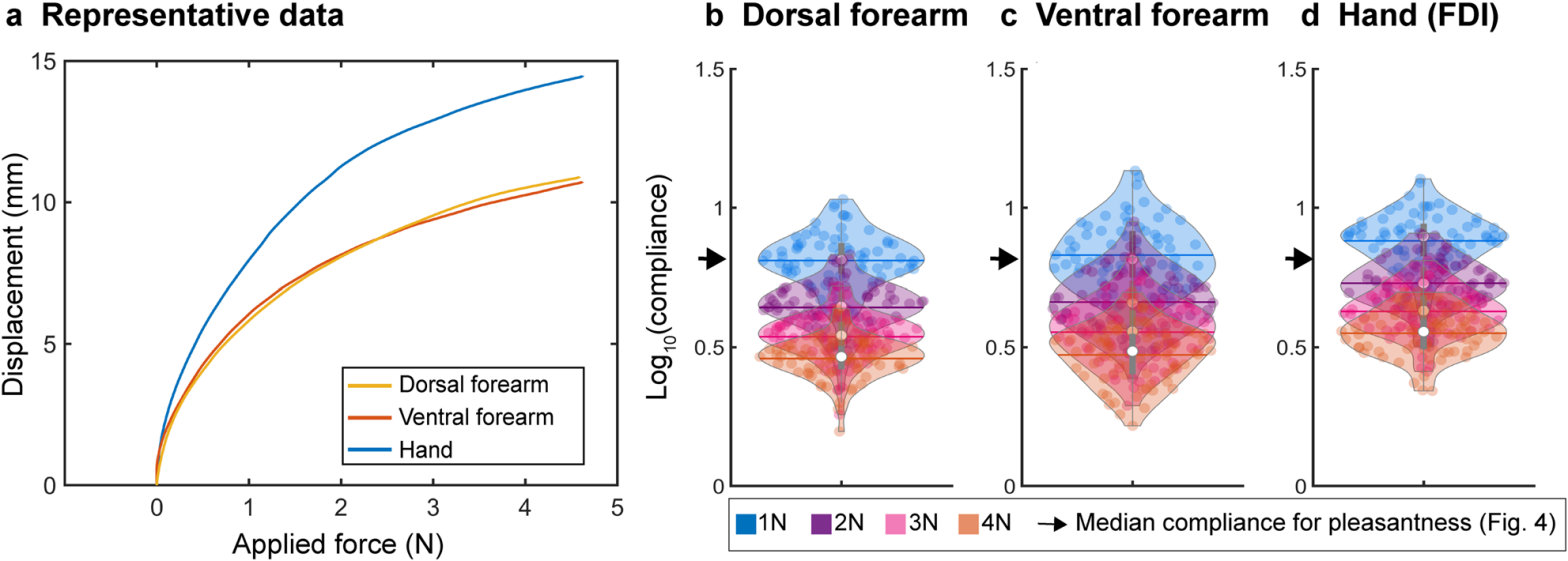
Distribution of compliance values for hands and forearms. We measured the forearm and hand compliance of 70 volunteers using the same setting as in Fig. 1b to measure body displacements with applied force. (**a**) Representative data. Unlike stimuli (Fig. 1b), the skin displacement did not increase linearly as a function of applied force. Compliance was determined as displacement per force at 1 N, 2 N, 3 N, and 4 N. (**b-d**) Distribution of compliance values for dorsal forearm, ventral forearm, and hand (n = 70) are shown as violin plots. The skin over the first dorsal interosseous (FDI) was used to measure hand compliance. Each dot represents the individual participant’s compliance at each force. The white circle, horizontal line, and bar indicate the median, mean, and box plot, respectively. Black arrows indicate the median compliance value for pleasantness (Fig. 4).

To test if the compliance providing the highest pleasantness corresponds to that of our bodies, we measured compliance of forearms and hands in 70 participants. We chose the forearm and arm because those are the body parts that we usually allow others to touch, regardless of the social network status^9,10^. Figure 6b–d shows the distributions of compliance for the dorsal forearm, ventral forearm, and hand (skin over the first dorsal interosseous muscle, FDI). Unlike the artificial stimuli, the human body consists of different tissues (e.g., muscle and bone) and hardens if pressed harder. Since the compliance changes depending on the force exerted (Fig. 6a), we measured the compliance when the skin is pressed by the probe at 1 N, 2 N, 3 N, and 4 N. The compliance ranged from 0.2 to 1.1 (on a base-10 logarithmic scale), more narrowly distributed than compliance values providing the highest pleasantness (Fig. 4a). Medians for the dorsal forearm were 0.81, 0.66, 0.56, and 0.49; for the ventral forearm 0.81, 0.65, 0.54, and 0.47; for the hand 0.90, 0.73, 0.63, and 0.56, at 1 N, 2 N, 3 N, and 4 N, respectively. Thus, the median compliance providing the highest pleasantness (0.81, Fig. 4a) was well within the compliance distribution for the hand and forearm and corresponded to the forearm’s compliance at 1 N. In summary, the compliance at the peak of the fitted function varied among the participants, while that of the group data was close to that of hand and forearm.

## Discussion

The present study had three main findings. First, the mean pleasantness was highest at around 5–7 mm/N of compliance. Second, pleasantness variances were minimum around the compliance that yielded the highest pleasantness. Finally, 5–7 mm/N correspond to the compliance obtained when the human forearm and hand were lightly touched.

We minimized the following confounding factors. First, we decreased stickiness by covering the stimuli with powder, and participants placed their finger in powder before touching the stimuli. Therefore, it is unlikely that our results were affected by stickiness. Finally, as in previous studies, we gave similar but separate instructions to participants for softness and pleasantness, with no hint of an association between the two. The supplementary experiment allowed us to exclude the participants who may have reported softness instead of pleasantness. Nevertheless, the pattern of results was highly similar even after excluding these participants. Thus, it is highly unlikely that our results are explained by participants in the pleasantness group reporting softness instead of pleasantness.

The present study confirmed our previous finding that compliance is a major contributor to perceived pleasantness^11^. However, these findings may seem contradictory to other previous findings. Previous studies examined the factors that contribute to affective touch by using affective adjectives^14,15,17^. In these studies, participants were asked to rate the touched objects by discriminative (e.g., soft and jagged) and affective adjectives (e.g., comfortable and thrilling). One study compared principal components analysis scores from ratings of discriminative adjectives to the corresponding scores from ratings of affective adjectives^15^. The component associated with valence was related to "roughness", but not to “formability.” However, this "roughness" component included the adjective "softness" as well as adjectives for roughness. In another study, "pleasantness" was related to the factor labeled "silken" that included "smooth" and "soft" attributes^17^. Thus, our present finding, together with the previous^11^, is not contradictory but rather complements existing research on tactile pleasantness by focusing on the relationship between softness and pleasantness.

Unlike our previous finding^11^, the present results show that softer objects do not necessarily provide higher pleasantness. We found that mean pleasantness reached a peak at around 5–7 mm/N and then showed a declining trend. Moreover, the variance of magnitude estimates tended to be low at higher pleasantness compliances; in other words, surfaces with compliance values at 5–7 mm/N tended to yield higher pleasantness than other surfaces *similarly* across participants. In addition, 5–7 mm/N corresponds to the compliance of hands and forearms, which are the most frequently touched body parts by others^9,10^.

These findings suggest that objects with similar compliance to a human hand and arm can be the most pleasant. Previous studies have shown that the most pleasant material properties are related to the physical properties of our body^20–22^. For example, the participants reported that objects at around 32 °C (typical human arm skin temperature) provide higher pleasantness than at other temperatures^22^. Kitada et al. (2012) showed that a dot pattern with inter-element spacing (500 um), close to the ridge-to-ridge distance of fingerprints^30^, was less unpleasant than patterns with greater inter-element spacing (up to 4 mm)^20^. In the present study, we extend these previous findings by showing that the most pleasant softness can correspond to the physical softness of our bodies. Our findings in conjunction with these previous findings support the hypothesis that tactile preferences may be adapted to the characteristics of the human skin.

The mean compliance that provides the highest pleasantness was slightly lower than that in our previous study (0.88 on the log scale)^11^. One plausible difference could be stimulus presentation. The present study employed active touch with one finger, whereas the previous adopted passive touch stimulation with three fingers. The participants used a higher maximum force per finger (9.9–10 N for one finger) in the present study than in the previous one (5 N or 20 N for three fingers). It is possible that the participants preferred slightly lower compliance when they could actively touch the surface and use their preferred force.

Our finding is in accord with the accumulating evidence that physical contact with the body parts of a familiar person can be beneficial^2^. For example, skin-to-skin contact with one's partner can decrease unpleasantness related to physical pain^7^ and social pain^8^. In a classic experiment, baby monkeys chose to stay with surrogate mothers wrapped in a soft cloth rather than surrogate mothers made of wires^1^. In humans, touching by caregivers can increase the number of smiles of the infants^31^ and reduce cortisol level of infants^32^, even though the caregiver provided neither emotional facial expression nor vocal cue. These effects may be caused because physical properties of our body can effectively cause a pleasant experience in caregiver-child interactions.

Though the mean peak compliance was approx. 5–7 mm/N, there were substantial individual differences. Six participants simply preferred softer objects, whereas two preferred harder objects. This result indicates that, although participants consider 5–7 mm/N as pleasant stimuli, compliance values yielding the most pleasantness vary across participants. There are several reasons why this could be the case. First, participants prefer softer objects simply because contact with hard objects can potentially cause more physical damage than contact with soft objects. Second, we frequently contact textiles softer than human bodies^33^. The contact with textiles can provide not only a softness perception but also that of smoothness and warmth, which are associated with pleasantness. Thus, the associated pleasantness of textiles may affect the preference for softer objects. Finally, this preference might be affected by food compliance, which can also be softer than body parts. It is possible that the relative contributions of these factors to our tactile preferences differ among individuals.

Previous neurophysiological studies have shown that C-tactile fibers, a unique set of unmyelinated afferent fibers located in hairy^34^ and glabrous^35^ skin, respond vigorously to slow, light stroking against the skin. C-tactile fibers may have the potential to elicit a pleasant subjective experience during gentle touch between individuals (the “social touch” hypothesis)^36^. However, since the participants in the present study applied normal but not tangential force to the stimuli, our result is unlikely to be explained by neural mechanisms involving C-tactile fibers. Rather, it is more reasonable to assume that mechanoreceptors innervated by Aβ afferents (e.g., SA-I afferents) contribute to perceived pleasantness as well as softness^37^. Indeed, a patient lacking Aβ afferents had difficulty in reporting conscious tactile experience, despite experiencing a faint and diffuse pleasant sensation^38^. Moreover, common brain networks involving common peripheral afferents are associated with the perception of roughness and softness^39,40^.

We did not observe a clear effect of compliance across stimuli on contact duration, while the maximum force applied to the surface decreased as a function of compliance. This finding is consistent with previous findings where participants systematically applied lower forces to more compliant objects in the haptic exploration of deformable surfaces (i.e., silicon rubbers)^41,42^. To examine an object's compliance, it is necessary to apply sufficient force to estimate the indentation. However, harder objects require greater force to indent them sufficiently. Thus, it is possible that participants might have adjusted the force so as to optimize the strategy for inferring compliance during contact.

There is one limitation worth noting. We limited the strategy of the participant's exploration of objects to pushing the stimuli because we planned to compare these results with our previous study^11^. Thus, further studies are necessary to examine if the pleasantness pattern remains the same when participants are allowed to freely explore the stimuli (e.g., applying tangential force to the surface).

In conclusion, the present study demonstrated that, despite individual differences in preferences, the median pleasantness of the deformable surface was highest between 5 and 7 mm/N, and the individual difference of pleasantness was lowest around this range of compliance. These values further correspond to the compliance of the human forearm and hand. This result supports the hypothesis that our preferences for touch are adapted to the physical properties of the human skin and body to satisfy the essential need for social bonds.

## Methods

### Participants

In this study, 118 participants were included (mean age = 20.5 years, range = 18 to 27 years old). Forty-eight right-handed volunteers (14 men and 34 women) participated in the psychophysical experiments. The number of participants was determined by our previous study^11^. Sample size estimation by BUCSS package in R^43^ indicated that 7 participants per group are sufficient to achieve a power of 0.95. We recruited 24 participants per group to match the number of participants between our studies^11^. In addition, 70 volunteers (66 right-handed and 4 left-handed; 17 men and 53 women) participated in the measurement of compliance for their own body parts. Body mass index (BMI) and fat ratio were measured by a weight scale (BC601, Tanita, Tokyo, Japan). BMI and fat ratio were 20.7 ± 3.2 and 24.4 ± 7.9 (mean ± SD), respectively.

Participants received either Research Participation (RP) credits or 5 Singaporean dollars (SGD) per 30 min of participation. All participants were free from finger and/or hand injuries, and handedness was determined according to the Fazio Handedness Inventory^44^, which is a modified version of the Edinburgh Handedness Inventory^45^. All participants provided written informed consent prior to beginning the experiment. The study protocol was reviewed and approved by the local Ethics Committee at Nanyang Technological University Singapore (PSY-IRB-2020–004; PSY-IRB-2020–035). All methods were carried out in accordance with the approved guidelines and the Declaration of Helsinki. Part of the study was preregistered at Open Science Framework (https://osf.io/4sqrd).

### Stimulus and stimulus presentation

We constructed eleven specimens made of non-carcinogenic polyurethane rubber (Katō Tech Co. Ltd., Kyoto, Japan; Fig. 1a), of which some were used in our previous study^11^. Each rubber stimulus consisted of a plastic box (9.5 cm length by 9.5 cm width by 2.5 cm depth) filled with polyurethane rubber. One side of each box was open to allow participants to touch the flat surface of the material directly. Baby powder (corn starch) was put on each surface to decrease the stickiness of the rubber as it can decrease pleasantness. The temperature of the stimuli was around 21 ºC.

We measured stimuli compliance using the same method as previously^11^. Briefly, each stimulus was placed on the platform of the compression tester (KES-G5, Katō Tech Co. Ltd., Kyoto, Japan); a flat-end cylindrical probe (1 cm^2^ area) was brought down to indent the surface of each stimulus until the applied force and deformation reached up to approx. 4.8 N and 12 mm, respectively. We fitted a linear function of the applied force to each stimulus data and obtained slope values for each stimulus (Fig. 1b). Compliances ranged from 0.09 to 43.8 mm/N. To measure the force applied by the participant and the duration of physical contact, the stimulus was placed on the center of a weighing machine (GX-8000, A&D Co. Ltd., Japan) connected to a laptop PC (CF-B10, Panasonic Corporation, Japan). The data sampling rate was 5 Hz.

### Design and procedures

We adopted an experimental design with one between-subject and one within-subject variable. We treated compliance as a within-subject variable, whereas instruction (softness and pleasantness evaluation) was designated as the between-subject variable to avoid the participants treating softness and pleasantness as identical. The ratio of biological sex was matched between the two instruction groups, as were their ages [*t*(46) = 0.07, *P* = 0.95]. The stimuli were presented in pseudo-random order and determined for each of the four repetitions per participant. The first repetition was considered practice and excluded from statistical analysis.

We adopted an absolute magnitude estimation procedure^11,20,26,27,46^. In one group, participants were asked to choose the number that best matched the pleasantness associated with each stimulus. In the other group, participants were instructed to choose the number that best matched the perceived softness of the stimulus. Participants could use any number (decimal, fraction, or whole number) as long as the number was above zero. Neither a modulus nor a standard was used. During practice repetition, all participants were familiarized with the magnitude estimation procedure.

The participants were blindfolded and sat in front of the table where the stimulus was placed. The experimenter, who sat at the opposite side, guided the participant's right hand over the surface of the stimulus on the weighing scale. As soon as the experimenter said "go," the participant was asked to push the stimulus with their right index finger up to three times. Once the participants finished touching the stimulus, they provided a magnitude estimate. The experimenter replaced the stimulus with another one for the next trial. In each trial, they put their index finger in baby powder to minimize the sensation of stickiness. The experiment was completed within 60 min.

### Analysis

We used a conventional procedure to analyze magnitude estimate scores^11,20,26,27,46^. The data averaged across three repetitions were normalized within each perceptual condition to eliminate possible biases due to the participants' use of different number ranges. This normalization procedure was performed by dividing each data point by the participant's mean and then multiplying that by the grand mean for the group (softness or pleasantness). Finally, the scores were logarithmically transformed (base 10) such that low-order polynomial functions were best fitted to the data. These transformed data were used for analyses. We used IBM SPSS Statistics (version 25.0, IBM Corp., Armonk, NY, U.S.) and MATLAB (2020a; MathWorks, Natick, MA, USA) for the following analyses.

Initially, we conducted Levene's test for equality of variances between all stimuli in each type with the correction of false-discovery (FDR) rate. We had planned to conduct repeated-measures ANOVAs at the preregistration stage. However, because Levene's tests showed significant differences in variances among stimuli, we conducted a linear mixed effect model (LMM) to account for different variances among stimuli (as exploratory analyses). Softness and pleasantness are qualitatively different perceptual dimensions. Thus, as in our previous studies^11,20^, we analyzed the magnitude estimates for each instruction separately. The subject factor (24) and 11 levels of compliance values were used for models with correlated residuals within random effects. In all analyses, we assumed compound symmetry with unequal variances (CSH) as covariance structure. We used the Bayesian information criterion (BIC) to select the best model when multiple models were constructed. After the LMM analyses, we evaluated the effect of instruction on magnitude estimates using compliance values providing the maximum estimates.

We also conducted the LMM analyses on contact duration and applied force that were obtained from the weighing machine. Since maximum force and contact duration can be compared between both instruction groups (pleasantness and softness), we added the instruction group and its interaction with compliance to the LMM.

### Measurements of body part compliance

We examined if high pleasantness compliance is related to that of body parts. Previous studies showed that forearms and hands are the body parts that we mostly allow others to touch^9,10^. Thus, we measured the compliance of the hand and dorsal and ventral sides of the forearm. Unlike stimuli, compliance depends on the force applied to the skin. Thus, we calculated compliance at 1, 2, 3, and 4 N and calculated each mean.

We used the same procedure as that used to measure stimuli compliance. Specifically, the participant was asked to place their right hand or arm on the compression tester platform (KES-G5, Katō Tech Co. Ltd.). They were also instructed to stay still and keep their forearm and hand relaxed during the measurement. A flat-end probe of 1 cm^2^ area was brought down to indent the surface of the subject’s forearm and hand until the applied force reached approx. 4.8 N. To measure compliance at 4 N, the upper limit of the deformation was increased up to 14 mm. The probe was applied to the ulnar part of the forearm where the skin was relatively flat. The points of the stimulation for the ventral and dorsal parts were 6.8 ± 1.7 (mean ± SD) cm and 8.0 ± 1.9 cm distal to the elbow crease, respectively. Likewise, the compression tester pushed the probe to the skin of the dorsal hand, the area over the first dorsal interosseous muscle (FDI) between thumb and index fingers. We chose these areas because the participants can keep the position still easily during the measurement.

## ﻿Supplementary Information

Supplementary Information.

## Data Availability

The datasets generated during and/or analyzed during the current study are available from the corresponding author on reasonable request.
