# Supplementary material for: Physical correlates of human-like softness elicit high tactile pleasantness
Source: Sci Rep. 2021 Aug 13;11:16510. doi: 10.1038/s41598-021-96044-w (PMC8363669; doi:10.1038/s41598-021-96044-w)
Supplement: Supplementary file 1 — Supplementary Information. [file 41598_2021_96044_MOESM1_ESM.docx]

**Supplementary Information**

Physical correlates of human-like softness elicit high tactile pleasantness

Ryo Kitada^1, 2^, Megan Ng^1^, Zheng Yee Tan^1^, Xue Er Lee^1^, Takanori Kochiyama^3^

1. Division of Psychology, School of Social Sciences, Nanyang Technological University, 48 Nanyang Avenue, SHHK-04-13, 639818, Singapore
2. Graduate School of Intercultural Studies, Kobe University, 1 Chome-2-1 Tsurukabuto, Nada Ward, Kobe, Hyogo, 657-0013, Japan
3. ATR-Promotions, Brain Activity Imaging Center, 2-2-2 Hikaridai Seika-cho, Sorakugun, Kyoto, 619-0288, Japan

**^*^Corresponding author:**

Ryo Kitada

School of Social Sciences, Nanyang Technological University, 48 Nanyang Avenue, 639818, Singapore. Tel: +65 6316 8935. E-mail: [ryokitada@ntu.edu.sg](mailto:ryokitada@ntu.edu.sg)

**Supplementary Table 1 Levene’s tests**

| Log_10_(Compliance) | | | | | | | | | | | |
| --- | --- | --- | --- | --- | --- | --- | --- | --- | --- | --- | --- |
|  | -1.06 | -0.39 | 0.02 | 0.35 | 0.53 | 0.66 | 0.77 | 0.91 | 1.23 | 1.28 | 1.64 |
| Pleasantness | | | | | | | | | | | |
| -1.06 |  | 0.2777 | 0.0646 | 0.0157 | 0.0052 | 0.0021 | 0.002 | 0.0052 | 0.4746 | 0.4746 | 0.7257 |
| -0.39 | 0.2777 |  | 0.3718 | 0.0937 | 0.0157 | 0.0052 | 0.0021 | 0.0157 | 0.7345 | 0.0794 | 0.4746 |
| 0.02 | 0.0646 | 0.3718 |  | 0.4086 | 0.0922 | 0.0204 | 0.0075 | 0.0794 | 0.3185 | 0.0157 | 0.1482 |
| 0.35 | 0.0157 | 0.0937 | 0.4086 |  | 0.4674 | 0.1755 | 0.0705 | 0.3718 | 0.1072 | 0.0055 | 0.0449 |
| 0.53 | 0.0052 | 0.0157 | 0.0922 | 0.4674 |  | 0.4746 | 0.2305 | 0.7554 | 0.0346 | 0.0021 | 0.0138 |
| 0.66 | 0.0021 | 0.0052 | 0.0204 | 0.1755 | 0.4746 |  | 0.556 | 0.7362 | 0.0157 | 0.002 | 0.0055 |
| 0.77 | 0.002 | 0.0021 | 0.0075 | 0.0705 | 0.2305 | 0.556 |  | 0.4707 | 0.0084 | 0.002 | 0.0036 |
| 0.91 | 0.0052 | 0.0157 | 0.0794 | 0.3718 | 0.7554 | 0.7362 | 0.4707 |  | 0.0296 | 0.0021 | 0.0124 |
| 1.23 | 0.4746 | 0.7345 | 0.3185 | 0.1072 | 0.0346 | 0.0157 | 0.0084 | 0.0296 |  | 0.1875 | 0.7257 |
| 1.28 | 0.4746 | 0.0794 | 0.0157 | 0.0055 | 0.0021 | 0.002 | 0.002 | 0.0021 | 0.1875 |  | 0.3267 |
| 1.64 | 0.7257 | 0.4746 | 0.1482 | 0.0449 | 0.0138 | 0.0055 | 0.0036 | 0.0124 | 0.7257 | 0.3267 |  |
|  | | | | | | | | | | |  |
| Softness | | | | | | | | | | | |
| -1.06 |  | 0.0769 | 0.0074 | 0.0003 | 0.0002 | 0 | 0 | 0 | 0 | 0 | 0 |
| -0.39 | 0.0769 |  | 0.3348 | 0.0134 | 0.0101 | 0.0003 | 0.0006 | 0.0003 | 0.0001 | 0.0003 | 0.0003 |
| 0.02 | 0.0074 | 0.3348 |  | 0.1351 | 0.0974 | 0.0028 | 0.0072 | 0.0026 | 0.0008 | 0.0026 | 0.0028 |
| 0.35 | 0.0003 | 0.0134 | 0.1351 |  | 0.8067 | 0.0675 | 0.1671 | 0.0634 | 0.0153 | 0.0622 | 0.0675 |
| 0.53 | 0.0002 | 0.0101 | 0.0974 | 0.8067 |  | 0.2169 | 0.4262 | 0.218 | 0.0974 | 0.2103 | 0.2267 |
| 0.66 | 0 | 0.0003 | 0.0028 | 0.0675 | 0.2169 |  | 0.5502 | 0.9044 | 0.6547 | 0.9726 | 0.9044 |
| 0.77 | 0 | 0.0006 | 0.0072 | 0.1671 | 0.4262 | 0.5502 |  | 0.578 | 0.1862 | 0.5359 | 0.5953 |
| 0.91 | 0 | 0.0003 | 0.0026 | 0.0634 | 0.218 | 0.9044 | 0.578 |  | 0.4525 | 0.9083 | 0.9726 |
| 1.23 | 0 | 0.0001 | 0.0008 | 0.0153 | 0.0974 | 0.6547 | 0.1862 | 0.4525 |  | 0.578 | 0.4525 |
| 1.28 | 0 | 0.0003 | 0.0026 | 0.0622 | 0.2103 | 0.9726 | 0.5359 | 0.9083 | 0.578 |  | 0.9044 |
| 1.64 | 0 | 0.0003 | 0.0028 | 0.0675 | 0.2267 | 0.9044 | 0.5953 | 0.9726 | 0.4525 | 0.9044 |  |

The value in each cell indicates the FDR-corrected p value of Levene’s tests (with false-discovery rate correction). Underlined numbers indicate significant effects.

**

**

**Supplementary Figure 1. Supplementary Experiment**

The purpose of the supplementary experiment was to find those participants who may have merely answered to the physical characteristics (e.g., compliance) as their perceived pleasantness. **a.** Stimuli were spherical plastic segments (4 mm diameter), varying from one to five. The participants in the softness-instruction condition estimated the number of spheres, whereas the participants in the pleasantness-instruction condition estimated the perceived pleasantness. Because all spheres have the same material and shape, pleasantness should not increase as a function of the number of spheres. Even though the contact with a few hemispheres can cause unpleasantness by exerting excessive force on a small skin area, pleasantness should not monotonically increase from 1 to 5 spheres.

**b.** Mean log_10_ normalised magnitude estimates of the number of spherical segments as a function of the number of spherical segments (n = 24).

**c.** Mean log_10_ normalised magnitude estimates of pleasantness as a function of the number of spherical segments (n = 24). We excluded participants whose magnitude estimates of pleasantness monotonically increased as a function of the number of spherical segments.

**

**

**Supplementary Figure 2. Supplementary analysis**

We analysed data of the main experiment after excluding 5 participants who showed a monotonic increase of perceived pleasantness as a function of the number of spherical segments in the supplementary experiment (Supplementary Figure 1). Mean log_10_ normalised magnitude estimates of pleasantness as a function of compliance.

**Analyses on deformable surfaces**

**1. Statistical analysis on contact duration**

We performed linear mixed model (LMM) analysis on the contact duration with a model that includes linear and quadratic trends of compliance, and group (two instructions: pleasantness and softness) as fixed-effect factors. The analysis revealed significant interaction between group and linear trend [*F*(1, 215.9) =5.95, *p* = 0.016].

Since we observed the interaction among factors, we then performed LMM analyses on each instruction group. The LMM analysis on data for the pleasantness instruction showed significant effects for linear and quadratic trends of compliance [*F*(1, 65.6) = 4.94, *p* = 0.03 for the linear trend; *F*(1, 165.8) = 6.54, *p* = 0.011 for the quadratic trend]. The fitted quadratic function was convex (the quadratic term was -0.09). Compliance value at the highest force was - 0.72 (on a logarithmic scale).

In other words, although the contact duration of the pleasantness instruction group appears largely constant across stimuli, the pleasantness data showed an inverted U-shaped pattern as a function of compliance with the peak where the compliance value was 0.2 mm/N. The same analysis on softness data showed a significant effect only for the quadratic trend of compliance [*F*(1, 208.2) = 13.6, *p* < 0.001] and indicates an inverted U-shaped pattern with a peak where the log_10_ compliance value was around 0.
